# Supplementary material for: Influenza vaccination coverage among emergency department personnel is associated with perception of vaccination and side effects, vaccination availability on site and the COVID-19 pandemic
Source: PLoS One. 2021 Nov 19;16(11):e0260213. doi: 10.1371/journal.pone.0260213 (PMC8604289; doi:10.1371/journal.pone.0260213)
Supplement: Attachment — Submitted filename: Response to Reviewers.docx [file pone.0260213.s001.docx]

**Journal Requirements:**

When submitting your revision, we need you to address these additional requirements.

1. Please ensure that your manuscript meets PLOS ONE's style requirements, including those for file naming. …

We have checked PLOS ONE´s style requirements again and hope everything is correct now.

2. Please provide additional details regarding participant consent …
We thank you for pointing this out. As the data were collected anonymously, self-administered and on a voluntary basis, no consent was obtained for this study. For more details please see lines 110-112.

3. Please provide the names of the participating EDs.

Thank you for this suggestion. We have made the change and added the names of the participating EDs. Please see lines 95-98.

4. please provide the date ranges when surveys were collected during the two seasons.

We apologize for not pointing this out. We have corrected the methods part and you can find the date ranges in lines 86-87 as well as in lines 90-91.

5. In your Data Availability statement, you have not specified where the minimal data set underlying the results described in your manuscript can be found. …

This observation is correct. An anonymized version of the dataset is available from zenodo.org

([https://doi.org/10.5281/zenodo.5164388](https://deref-gmx.net/mail/client/qRtn-W-bF3w/dereferrer/?redirectUrl=https%3A%2F%2Fdoi.org%2F10.5281%2Fzenodo.5164388)). "

**Reviewers' comments:
Reviewer 1:**

Abstract, line 27: The extremely low response rate in [2020-2021](tel:20202021) is a threat to the validity of your findings and should be noted in the limitations section (which is missing from the paper).

We thank the reviewer for pointing this out. We have added a limitation section at the end of the discussion, which, among others, concerns to the response rate in 2020/2021. Please see lines 339-342.

Abstract, line 33: The pandemic is not a factor that differs across the study population; this could be tweaked to say something like "the effect of the pandemic on influenza vaccination intentions" or similar.

We agree and have updated this sentence. Please see line 31.

Introduction, line 52: Usually influenza vaccination is also recommended for those at increased risk of complications from influenza disease…

Thank you for this important observation. We have changed this section. Please see lines 54 and 60-61.

Methods, line 76: Given that the article presents a comparison with findings in the COVID-19 pandemic context and the earlier findings, it would be useful to include a brief note … about the impetus for the initial [2016-2017](tel:20162017) survey…

We have revised the text to address your concerns and explained in more detail the impetus for the first survey in 2016/2017 and the linkage to the second survey. Please see lines 85-93**.**

Methods, line 77: Are these 14 EDs the universe of EDs in Bavaria? If not, how many are there/what percentage is represented by the 14 invited, and how were they selected for recruitment?

We thank the reviewer for pointing this out. We added this information to the limitations at lines 337-339.

Methods, lines 89-99: The sample selection requires substantial clarification. … Also, please provide more detail on how specifically the surveys were disseminated ...

We agree and have corrected the sample recruitment part with a more detailed explanation of the process. Please see lines 113-117. We also revised the text to include your concerns about multiple surveys in the new limitations section. Please see lines 343-345.

Results, Table 1: Please change "vaccinated because of COVID-19" to "intending to be vaccinated because..." … you might consider reformatting it or even breaking it up into multiple tables …

We have made the changes and thank the reviewer for these suggestions. Please see lines 165-171.

Results, line 148: The fact that the comparisons of unvaccinated with vaccinated persons include only data from [2020-21](tel:202021) should be noted at every point in the manuscript or tables/figures where these comparisons are made.

We have taken note of this important observation and added the note in the certain text passages. Please see lines 29,36,199,204, 215,220,223,224,239,327.

Results, line 157: Higher risk relative to what, or whom

We agree that this information is missing and added the comparison group in lines 190-191.

Results, line 160: …the figure titles need to be more descriptive so that they can be interpreted without reading the text. I would also suggest including in the figure title that the figures compare [2016-17](tel:201617) to [2020-21](tel:202021) and compare unvaccinated to vaccinated persons.

We´ve revised the figure titles to become more descriptive and changed the figure titles as suggested. Please see lines 194-201, 210-217, 233-240.

Results, line 169: How was this list of possible vaccine side effects selected… Also, how were these presented in the survey? Listing very serious and extremely rare side effects alongside mild and common side effects could potentially bias respondents' selections.

We thank the reviewer for pointing this out. The list of side effects was loosely built on previous studies, which we added in the method part (query and variables) in lines 129-130. The order of the side effects listing in the paper is almost the same than in the questionnaires. Also, we thank you for your concern regarding the alongside listing of mild, extreme and almost nonexistent side effects listings. We are aware that some effects are very rare. However, this was done intentionally to determine whether effects were partially overestimated.

Results, line 184: … did you attempt to combine data across seasons to see if the same associations with vaccination status persist? …

This observation is correct and the comparison would be interesting, but in 2016/2017 season, the items pertaining to the SARS-CoV-2 pandemic was not included so this variable would be missing. We explained that in more detail in lines 148-150.

Discussion, general comment: … Areas that seem redundant and could be deleted entirely or reduced to a single sentence include lines [213-217](tel:213217) and [223-230](tel:223230).

We thank the reviewer for this suggestion. We agree and have changed this part of the discussion. Please see lines 263-268 and 282-285.

Discussion, lines [201-206](tel:201206): This should be noted in detail in the introduction as context and then briefly restated here in the discussion if needed.

We´ve corrected this part of the discussion as we agree on the reviewers suggestion. Please see lines 54-60, 64-67 and 249-254.

Discussion, lines [207-212](tel:207212): Some of this is already in the introduction and could be deleted…

We revised the discussion and introduction part and thank the author for this suggestion. Please see lines 51-52, 243-249 and 255-259.

Discussion, lines [231-234](tel:231234): It would be helpful here to include some suggested actions to take based on these findings …

We thank the reviewer for this comment and have added a section with suggested actions for a higher vaccination rate. Please see lines 297-308.

Discussion, lines [236-239](tel:236239): … I would recommend a discussion paragraph talking about differences and similarities over time, and then a separate paragraph discussing differences observed by vaccination status in [2020-2021](tel:20202021).

We agree that this part of the discussion was confusing. We have revised the structure of the discussion as the reviewer suggested. Please see lines 271-290 and 310-317.

Discussion, lines [249-250](tel:249250): I would create a separate paragraph focusing on the ethics questions/findings and implications for pandemic preparedness, vaccination promotion, etc.

We thank the reviewer for this pleasing assessment. We have extended the ethical findings sections. Please see lines 325-330.

Discussion, line 259: As noted above, the manuscript lacks a limitations section; one should be added here.

We apologize for the missing limitation section, which was now included in the last part of the discussion. Please see lines 337-345

Conclusion, line 261: I did not see a measure of awareness in this study; this should be rephrased to reflect what was actually asked in the survey.

We have corrected the term and changed it into the assessment of influenza as a more serious risk. Please see lines 348-349.

Conclusion, lines [263-264](tel:263264): … I strongly suggest adding to your discussion a supporting paragraph that discusses the change in onsite vaccine offering over time…

We thank the reviewer for his important notes and added some reference papers to our study. Please see lines 290-293.

Conclusion, lines [266-267](tel:266267): … I would not term these "most beneficial". I suggest recommending this type of effort in conjunction with onsite offering of influenza vaccine as discussed above.

We have changed the text according to the reviewer´s suggestion. Please see line354-355

Figures 2-4: The labels on individual figure entries need to be edited to be more specific as many are not interpretable without a copy of the survey instrument …

We agree to the reviewer´s comment and described the items on the figures in more detail. Please see Figures 2-4.

**Reviewer 2:**
I have three questions :
1- Could the authors please elaborate on the fact that vaccination was not available on sit everywhere? …

Thank you for your questions, which we would like to address in more detail. In Germany, vaccinations are generally free of charge for hospital employees. However, vaccinations do not take place exclusively in the ED, but can also be given, for example, via an appointment with the company doctor.

2- Do they authors have the [2020/2021](tel:20202021) national vaccination coverage in physicians and nurses as a comparator ?

Unfortunately, the requested data is not yet available.

3- Could you precise others categories than administrative personnel in the non physicians or nurses people ? Did you survey other personnels in contact with patients such as cargivers known for their low vaccine coverage?

We thank the reviewer for this important question. The others category only includes non medical staff (mainly administrative). Caregivers were not included in this survey, only registered nurses.
